# Association of Endometrial *HAND2* Expression with β_2_-Glycoprotein I Antibodies in Recurrent Pregnancy Loss

**DOI:** 10.3390/biomedicines14071560

**Published:** 2026-07-12

**Authors:** Madina Khalmirzaeva, Gulzhakhan Omarova, Almagul Kurmanova, Gaukhar Kurmanova, Gaini Anartayeva, Zhamilya Zhankina, Aidana Tulesheva, Ainura Veliyeva, Botakuz Jarikova, Alfiya Dzheksembekova

**Affiliations:** 1 Faculty of Medicine and Healthcare, Farabi University, 71, Al Farabi Avenue, 050040 Almaty, Kazakhstan; madinakhalmirzaeva7@gmail.com (M.K.); gaukhar.kurmanova@kaznu.kz (G.K.); ecomed_gaini@mail.ru (G.A.); aidana.tulesheva@mail.ru (A.T.); ainura.veliyeva@gmail.com (A.V.); djarikova_bota@mail.ru (B.J.); elf_ernazarovna@mail.ru (A.D.); 2 Department of Biology, Faculty of Natural Sciences, Friedrich-Alexander University, Erlangen-Nürnberg, Schlossplatz 4, 91054 Erlangen, Germany; zhamilya.zhankina@fau.de

**Keywords:** recurrent pregnancy loss, β_2_-glycoprotein I, *HAND2*, endometrial receptivity, endometrial transcriptome

## Abstract

**Background**: Recurrent pregnancy loss (RPL) is frequently linked to antiphospholipid antibodies, yet many affected women are seronegative for the classical (Sydney) criteria antibodies. In a previously characterized cohort, an extended antiphospholipid antibody panel was positive in 79% of women with RPL, with the strongest associations for antibodies against prothrombin, annexin V and β_2_-glycoprotein I. Whether this association reflects molecular endometrial dysfunction was unclear. **Objective**: To examine endometrial expression of ten decidualization- and inflammation-related genes in women with RPL, stratified by APL status, focusing on the dominant antibody specificity: anti-β_2_-glycoprotein I. **Methods**: Thirty-nine women with RPL (≥2 losses), a subsample of a published 100-patient cohort, underwent mid-luteal Pipelle endometrial biopsy followed by RT-PCR for *C4BPA*, *CXCL1*, *HAND2*, *HPRT1*, *IFNG*, *IL15*, *IL8 (CXCL8)*, *MMP10*, *TNC* and *VEGFB*. Antibodies were measured by antiphospholipid 10 Dot line immunoblot (Generic Assays, Germany). The analysis used Mann–Whitney U, Fisher’s exact test, Benjamini–Hochberg correction and ROC analysis. **Results**: Antibody prevalence in the subsample matched the parent cohort (Pearson r = 0.999). Of ten transcripts, only *HAND2* showed a nominally significant reduction in APL+ women (median ΔCt −2.04 vs. −1.30; *p* = 0.037; rank-biserial r = −0.47); this signal did not survive Benjamini–Hochberg correction (q = 0.372). The signal was specific to anti-β_2_GPI (*p* = 0.064; AUC 0.700), not to annexin V or prothrombin. A cut-off of *HAND2* ΔCt ≤ −1.60 identified APL+ status with 73% sensitivity, 78% specificity and OR 9.62 (95% CI 1.64–56.4; *p* = 0.015). *HAND2* clustered with *IL15* (ρ = 0.65) and *VEGFB* (ρ = 0.70). **Conclusions**: Anti-β_2_-glycoprotein I antibodies, the dominant APL specificity in RPL, are associated with reduced endometrial *HAND2* expression. This exploratory data outline a candidate molecular pathway linking extended antiphospholipid serology to impaired endometrial receptivity.

## 1. Introduction

Recurrent pregnancy loss (RPL), defined as two or more clinical losses, affects 1–3% of women of reproductive age and remains a diagnostically and psychologically challenging condition [1,2]. Up to 40–50% of cases are classified as “unexplained” after standard work-up according to the 2023 ESHRE guideline [1], reflecting the existence of yet unrecognized pathogenetic mechanisms.

Among the immune-mediated causes of RPL, antiphospholipid syndrome (APS) occupies a central place. The classical laboratory diagnosis of APS, according to the Sydney criteria [3] and the 2023 ACR/EULAR update [4], requires detection of at least one of three antibodies—lupus anticoagulant, anti-cardiolipin or anti-β_2_-glycoprotein I—confirmed twice with an interval of ≥12 weeks. Nevertheless, a substantial proportion of patients with a clinical APS phenotype, including RPL, remain seronegative for these criteria antibodies [5,6,7], which gave rise to the concept of seronegative obstetric APS.

A recently published study [8] assessed the diagnostic value of an extended panel of 10 antiphospholipid antibody markers in 100 women with recurrent miscarriages and 20 healthy controls with an uncomplicated obstetric history. The antiphospholipid antibodies were detected in 79.0% of women with RPL and were significantly associated with the condition. Three specificities reached statistical significance in univariate analysis: antibodies against prothrombin (OR 11.1; 95% CI 1.8–68.0; *p* = 0.022), antibodies against annexin V (OR 4.28; 95% CI 1.18–15.6; *p* = 0.023) and antibodies against β_2_-glycoprotein I (OR 3.31; 95% CI 1.18–9.28; *p* = 0.019) [8]. A composite immunological score showed moderate discriminative ability (AUC = 0.701; *p* = 0.005). These data supported the hypothesis that non-criteria antiphospholipid serology represents a clinically relevant, yet underrecognized, component of the diagnostic evaluation of RPL. The conceptual rationale for an extended panel of 10 antiphospholipid antibodies and their pathophysiological role in pregnancy complications is provided in a systematic review [9].

The altered expression of endometrial receptivity genes, particularly immune response genes, is one of the causes of reproductive disorders such as RPL and RIF [10]. The molecular mechanisms of obstetric APS, including complement activation, endothelial damage, and trophoblast dysfunction, may lead to impaired decidualization and be associated with genetic or epigenetic factors. However, within the “immunothrombosis” model, empirical confirmation using non-criterial antiphospholipid serology was lacking. Successful implantation requires coordinated stromal decidualization of the endometrium, driven by progesterone, cAMP, and a network of transcription factors [11]. Among them, the basic helix–loop–helix factor *HAND2* (Heart and Neural Crest Derivatives expressed 2) is a master regulator of stromal decidualization: it suppresses stromal FGF expression, dampens proliferative epithelial ERK signaling and enables decidual reprogramming [12,13]. The conditional inactivation of *HAND2* in the murine uterus produces a non-receptive endometrium and embryonic lethality [12]. In humans, reduced endometrial *HAND2* expression has been described in endometriosis-associated infertility [13,14], in unexplained implantation failure [15], and in the setting of *HAND2*-promoter hypermethylation in women with RPL [16]. To our knowledge, the *HAND2* axis has not previously been explored in the context of antiphospholipid serology or in relation to the dominant anti-β_2_GPI specificity.

The present study is a molecular extension of our previous serological work [8]. Its aim is to describe the expression profile of ten endometrial genes spanning decidualization (*HAND2*, *IL15*, *VEGFB*), inflammation (*CXCL1*, *IL8*, *IFNG*, *MMP10*, *C4BPA*) and extracellular matrix remodeling (*TNC*) axes in a subsample of 39 women with RPL who underwent endometrial biopsy and to determine which specific antibody specificity associates with any observed transcriptional shift. The accompanying markers were selected to span three biological axes implicated in endometrial receptivity and in the immunothrombosis model of obstetric APS. Within the decidualization axis, *IL15* was included as a key regulator of uterine natural killer cells that support spiral-artery remodeling and trophoblast invasion [8], and *VEGFB* as a mediator of endometrial angiogenesis required for receptivity [8,9]. The inflammatory axis comprised the neutrophil chemokines *CXCL1* and *IL8*, whose endometrial up-regulation reflects a pro-inflammatory, receptivity-impairing milieu [10]; the Th1 cytokine *IFNG*, whose excess is linked to implantation failure and pregnancy loss [8]; and *C4BPA*, a complement regulator that connects the panel to the complement-mediated injury characterizing obstetric APS [8,9,10]. Finally, the extracellular-matrix axis was represented by *MMP10*, involved in endometrial tissue remodeling and trophoblast invasion [10], and *TNC* (Tenascin-C), an injury-associated matrix glycoprotein up-regulated in a progesterone-resistant endometrium [9]. These transcripts were pre-specified to capture decidualization, inflammation and matrix remodeling around the central candidate *HAND2*.

## 2. Materials and Methods

### 2.1. Study Design

The present study is a prospective molecular sub-analysis of our previously described cohort [8]. Of the 100 women with RPL enrolled in the parent study, 39—who met an additional criterion of having a mid-luteal Pipelle endometrial biopsy within the same diagnostic visit—formed the molecular subsample of the present study. All participants provided separate written informed consent for molecular analysis of biological material.

Inclusion criteria (identical to the previous study [8]): reproductive age 18–42 years; ≥2 documented spontaneous pregnancy losses before 24 weeks of gestation; regular menstrual cycle (24–35 days); absence of acute illness at enrollment. Additional criterion for the present sub-analysis: availability of a Pipelle endometrial biopsy obtained 7–9 days after the LH surge.

Exclusion criteria (identical to the previous study [8]): chromosomal abnormalities in either partner confirmed by karyotyping; clinically relevant somatic comorbidities (uncontrolled diabetes, decompensated thyroid disease, chronic kidney disease with eGFR < 60 mL/min/1.73 m^2^, chronic liver disease, malignancy, documented systemic autoimmune disease meeting classification criteria); confirmed congenital uterine anomalies; acute infection at enrollment.

### 2.2. Endometrial Biopsy and Sample Processing

Pipelle endometrial biopsies were obtained on days 7–9 after the LH surge (urinary LH test; LH+7 ± 1), corresponding to the mid-secretory window of implantation. Tissue was immediately placed in RNAlater (Thermo Fisher Scientific, Waltham, MA, USA), stored at +4 °C for up to 24 h and then transferred to −80 °C. Total RNA was extracted using a column-based kit [RNeasy Mini Kit (Qiagen, Hilden, Germany) or equivalent] with on-column DNase I treatment. RNA concentration and 260/280 ratio were assessed on a NanoDrop ND-1000 spectrophotometer (Thermo Fisher Scientific, Waltham, MA, USA); RNA integrity was verified with a threshold of RIN ≥ 7.0.

### 2.3. Real-Time RT-PCR

cDNA was synthesized from 500 ng of total RNA (High-Capacity cDNA Reverse Transcription Kit, Applied Biosystems, Waltham, MA, USA). Quantitative PCR was performed on a StepOnePlus Real-Time PCR System (Applied Biosystems) using TaqMan Gene Expression Assays for ten transcripts, selected to capture three biologically distinct endometrial axes:

(i) Decidualization and receptivity—*HAND2* (master decidualization transcription factor), *IL15* (uterine NK-cell support cytokine), *VEGFB* (angiogenesis);
(ii) Inflammation—*CXCL1* and *IL8* (CXCL8) (neutrophil chemokines), *IFNG* (Th1), C4BPA (complement regulation);
(iii) Extracellular matrix remodeling—*MMP10* (matrix metalloproteinase 10) and *TNC* (Tenascin-C, an injury-associated glycoprotein).

*HPRT1* was treated here as one of the assayed panel transcripts. Normalization was performed against two reference genes, *GAPDH* and *YWHAZ*, using their geometric mean. Reactions were run in duplicate, with no-template and no-reverse-transcription controls. Cycling conditions were 95 °C for 10 min, followed by 40 cycles of 95 °C for 15 s and 60 °C for 60 s. Relative expression was calculated by the comparative ΔΔCt method, with modifications as described by Königshoff et al. (2009) [17]; values are reported as relative expression (ΔCt) on a log_2_ scale, with more negative values indicating lower transcript abundance relative to the pooled calibrator.

### 2.4. Antiphospholipid Serology

Antiphospholipid antibody detection was performed by antiphospholipid 10 Dot line immunoblot (REF 5012; GA Generic Assays GmbH, Blankenfelde-Mahlow, Germany; IVD/CE-marked), described in detail in our earlier methodological review of antiphospholipid serology [9]. The kit simultaneously detects IgG and IgM antibodies against ten phospholipid antigens and protein cofactors: cardiolipin, phosphatidic acid, phosphatidylcholine, phosphatidylethanolamine, phosphatidylglycerol, phosphatidylinositol, phosphatidylserine, annexin V, β_2_-glycoprotein I and prothrombin. Assays were performed according to the manufacturer’s protocol with a final qualitative visual assessment of staining intensity relative to the control line. In addition to the immunoblot panel, all participants were tested for the classical Sydney criteria markers: lupus anticoagulant by the standard two-step coagulometric protocol and anti-β_2_GPI IgG/IgM by chemiluminescent immunoassay. The testing was performed once at enrollment; the limitations of this approach are discussed below.

### 2.5. Statistical Analysis

The analysis was performed in IBM SPSS Statistics v26.0 (IBM Corp., Armonk, NY, USA), consistent with the parent study [8]. Continuous variables are presented as median and interquartile range—Md [IQR]—given non-normal distribution (Shapiro–Wilk test); categorical variables as *n* (%). Between-group comparisons used Mann–Whitney U for continuous and Fisher’s exact test for categorical variables. Effect size for non-parametric comparisons is expressed as rank-biserial correlation r. Correlations between continuous variables are reported as Spearman ρ. Multiplicity over the 10-gene panel was controlled by the Benjamini–Hochberg procedure (FDR = 0.10). The discriminative ability of *HAND2* was assessed by ROC analysis with area under the curve (AUC) and 95% CIs (bootstrap, 2000 resamples); the optimal cut-off was determined by Youden’s J. Statistical significance was set at two-sided *p* < 0.05.

The sample size (*n* = 39) provided ≥80% statistical power (two-sided α = 0.05) to detect a Cohen’s d ≥ 0.85 under the observed 9:30 group distribution. Analyses are framed as hypothesis-generating, with priority given to effect sizes and confidence intervals over *p*-values alone.

### 2.6. Ethical Approval

The molecular analysis was performed under the original ethics approval of the Ethics Committee of the Kazakhstan Medical University “Higher School of Public Health” (IRB-70-2023; approval date 6 May 2025), under the same protocol as the parent study. All participants signed informed consent for the use of biological material in accordance with the Declaration of Helsinki.

## 3. Results

### 3.1. Cohort Characteristics and Representativeness

Of the 100 patients with RPL described in the previous study [8], 39 (39.0%) underwent Pipelle endometrial biopsy followed by RNA extraction of sufficient quality (RIN ≥ 7.0) and formed the molecular subsample of the present study. Baseline clinical characteristics of the subsample are presented in Table 1: median age 32 years (IQR 30–35), median BMI 22 kg/m^2^ (IQR 21–25), median 2 (IQR 2–4) pregnancy losses—consistent with the profile of the parent cohort.

The prevalence of antiphospholipid antibodies in the subsample (Table 2) was nearly identical to that in the parent 100-patient cohort: anti-β_2_GPI 74.4% vs. 73.0%; anti-annexin V 41.0% vs. 43.0%; anti-prothrombin 23.1% vs. 21.0%.

The distribution is closely reproduced; the three antibodies with the highest OR in the parent study [8] are listed first; the sum of any antibody is shown at the bottom.

**Figure 1:**
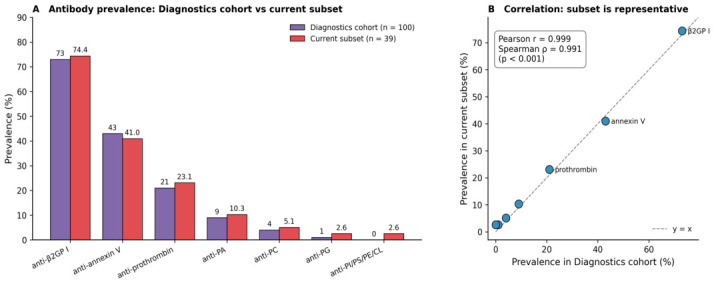
Representativeness of the present subsample relative to the published 100-patient cohort [8]. (**A**) Comparison of antiphospholipid antibody prevalence in the parent cohort (*n* = 100) and in the present subsample (*n* = 39). (**B**) Correlation of antibody prevalences between the two groups (Pearson r = 0.999; Spearman ρ = 0.991) with the y = x identity line; the subsample almost exactly reproduces the serological profile of the parent cohort.

The correlation between prevalences across all ten antibodies was Pearson r = 0.999, Spearman ρ = 0.991 (Figure 1).

This high consistency confirms the representativeness of the subsample relative to the parent cohort [8] and ensures continuity of the serological signal for the molecular investigation.

### 3.2. Endometrial HAND2 Expression Is Reduced in Antiphospholipid-Positive Women

Of the ten panel transcripts (Table 3), *HAND2* was the only gene that discriminated antibody-positive from antibody-negative women at a nominal significance level (uncorrected *p* = 0.037).

**Figure 2:**
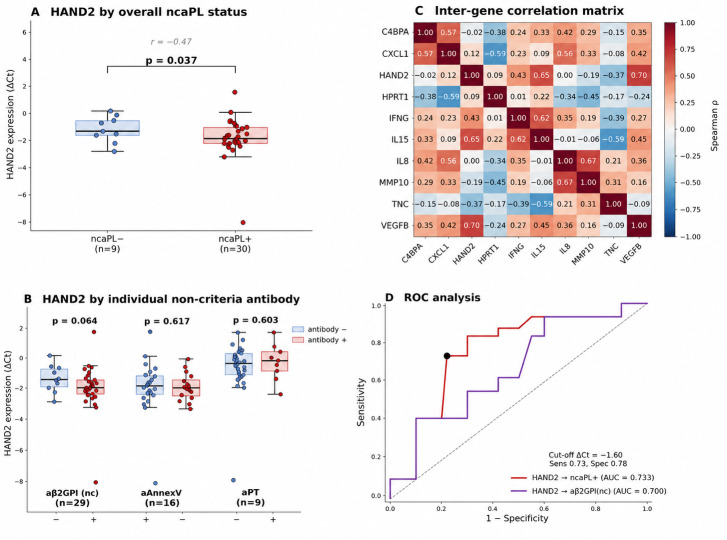
Main result. (**A**) *HAND2* expression (ΔCt) in ncAPL− (*n* = 9) and ncAPL+ (*n* = 30) women; *p* = 0.037; r = −0.47. (**B**) *HAND2* by each of the three antibodies associated with RPL; the signal is attributable specifically to anti-β_2_GPI. (**C**) Spearman correlation matrix for the 10-gene panel showing two coherent modules: decidualization (*HAND2–IL15–VEGFB*, ρ = 0.45–0.70) and inflammation (*IL8–MMP10–CXCL1–C4BPA*, ρ = 0.33–0.67). (**D**) ROC curves for *HAND2* as a predictor of ncAPL+ and anti-β_2_GPI+ status; the optimal cut-off ΔCt = −1.60 is indicated.

The median *HAND2* ΔCt was −2.04 [IQR −2.45; −1.56] in ncAPL+ versus −1.30 [IQR −1.56; −0.48] in ncAPL− women (Mann–Whitney U *p* = 0.037; rank-biserial correlation r = −0.47, corresponding to a medium-to-large effect). *VEGFB* showed a co-directional but weaker signal (median ΔCt −3.19 vs. −2.89; *p* = 0.188; r = −0.30); the remaining eight transcripts had absolute |r| ≤ 0.18 (Table 3, Figure 2A).

After a stringent Benjamini–Hochberg correction across the ten transcripts, the *HAND2* signal did not survive the FDR threshold (q = 0.372). This result is interpreted in light of the limited subsample size and within the coherent biological framework discussed below (Section 4); taken together, the effect size (r = −0.47), the consistent direction of effect across decidualization-related transcripts, and the large OR obtained in the categorical analysis (below) support a biologically plausible, hypothesis-generating association between *HAND2* and ncAPL status that warrants confirmation in larger, independent cohorts.

### 3.3. The HAND2 Signal Is Specific to Antibodies Against β_2_-Glycoprotein I

To localize the *HAND2* association within the ncAPL panel, we stratified *HAND2* expression by each of the three antibodies that were associated with RPL in the parent study [8] (Table 4, Figure 2B).

The association was specific to anti-β_2_GPI: median *HAND2* ΔCt −2.02 [IQR −2.46; −1.55] in anti-β_2_GPI+ vs. −1.39 [IQR −1.94; −0.48] in anti-β_2_GPI− women (*p* = 0.064; r = −0.40; AUC 0.700). Antibodies against annexin V (*p* = 0.617) and prothrombin (*p* = 0.803) did not show any signal with *HAND2* (|r| ≤ 0.10).

This pattern is biologically meaningful: of the three antibodies associated with RPL in a serological study [8], only anti-β_2_GPI—directed against the dominant antigenic cofactor of APS [18,19]—is associated with a molecular marker of endometrial decidualization. Annexin V and prothrombin antibodies act mainly at the level of coagulation and trophoblast invasion [20,21], whereas β_2_-glycoprotein I—uniquely among the three—is exposed on anionic phospholipid surfaces of the decidual stroma [22] and signals via TLR4 in decidual cells [23], providing a direct link to the progesterone-dependent transcriptional program of *HAND2* [24].

### 3.4. ROC Analysis and a Diagnostic HAND2 Cut-Off

*HAND2* expression discriminated antibody-positive from antibody-negative women with an AUC of 0.733 (95% CI 0.50–0.92) and anti-β_2_GPI+ status with an AUC of 0.700 (95% CI 0.47–0.90; Figure 2D). Youden’s J identified an optimal cut-off of ΔCt = −1.60: women with *HAND2* ΔCt ≤ −1.60 showed 73% sensitivity and 78% specificity for extended-panel antibody-positive status, with an odds ratio of 9.62 (95% CI 1.64–56.4; Fisher’s exact *p* = 0.015; PPV 92%, NPV 47%).

The overall expression-profile picture (Figure 2) shows that (A) *HAND2* is reduced in extended-panel antibody-positive at a nominal significance level and with a large effect size; (B) the signal is attributable specifically to anti-β_2_GPI; (C) the inter-gene correlation matrix reveals two coherent modules—a decidualization module (*HAND2–IL15–VEGFB*) and an inflammatory module (*IL8–MMP10–CXCL1–C4BPA*); and (D) *HAND2* shows potential value as a candidate biomarker of antibody-positive status.

### 3.5. Patient-Level Structure of Transcriptional Clusters

**Figure 3:**
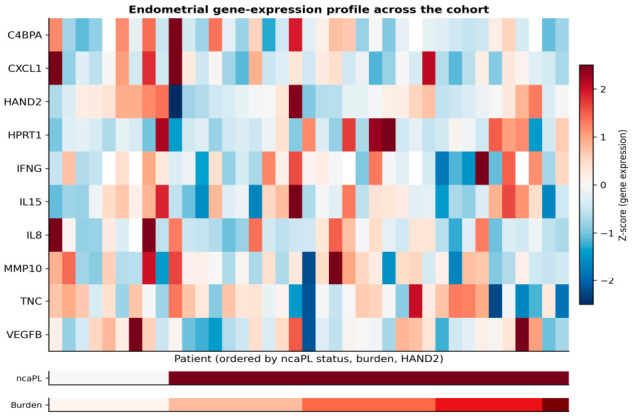
Patient-level heat map of endometrial expression for the ten panel genes (within-gene z-score; blue—below, red—above the mean). Patients are ordered left to right by ncAPL status, antibody burden and *HAND2* value. The bottom strips show antibody status (0/1) and the number of positive ncAPL specificities (0–4). A separation of the “cold” decidualization pattern (*HAND2/IL15/VEGFB*) in antibody-positive patients is visible.

A patient-level expression heat map (Figure 3), ordered by antibody status and *HAND2* value, visually illustrates the separation of transcriptional patterns: antibody-positive women show a predominantly “cold” pattern in the decidualization module (*HAND2*, *IL15*, *VEGFB*), accompanied by elevated TNC. This dual pattern, reduced decidualization markers together with elevated Tenascin-C, an injury-associated matrix glycoprotein, is consistent with a progesterone-resistant state of the endometrium under an immune-mediated load.

## 4. Discussion

### 4.1. Main Findings in the Context of the Previous Serological Work

The present study extends our previous serological findings [8] to the molecular level. Three observations deserve particular emphasis. First, the subsample of 39 patients with endometrial biopsy is serologically indistinguishable from the full 100-patient cohort (Pearson r = 0.999 between the prevalences of all ten antibodies), which ensures continuity with the parent study and allows the molecular findings to be interpreted within the same serological framework. Second, of the ten panel transcripts, *HAND2* is the only gene that is significantly reduced in antibody-positive women. Third, this signal is attributable specifically to antibodies against β_2_-glycoprotein I, and not to annexin V or prothrombin—biologically consistent with the specific role of β_2_GPI as the dominant antigenic cofactor with direct access to the decidual stroma [18,19,22,23].

### 4.2. Molecular Mechanism: From Anti-β_2_GPI to HAND2

In the “immunothrombosis” model, which combines complement activation (C3/C4) induced by antiphospholipid antibodies, endothelial injury, Th1/Th17 cell recruitment, and trophoblast dysfunction, these factors represent a single cascade. The present data on reduced *HAND2* expression in anti-β_2_GPI-positive women add an endometrial component to this model. A biologically plausible mechanism links anti-β_2_GPI to suppression of *HAND2* via the progesterone-resistance axis. Anti-β_2_GPI antibodies trigger TLR4/MyD88 signaling and a complement-mediated (C5a) inflammatory cascade in decidual cells with NF-κB activation [23,25]. NF-κB is a recognized antagonist of progesterone receptor signaling [26]; because HAND2 is a progesterone-dependent transcript [12], its suppression under anti-β_2_GPI-induced NF-κB activation follows logically from this chain. The fact that in our data the *HAND2* signal is specific to β_2_GPI—and not to annexin V or prothrombin—supports a β_2_GPI-domain-specific signaling axis on the decidual stroma as the underlying mechanism.

The predominance of anti-β_2_GPI in our extended panel (74% in the subsample, 73% in the parent cohort [8]) is explained by three properties of β_2_-glycoprotein I. First, β_2_GPI is the true antigenic cofactor of “anti-cardiolipin” antibodies [18,19]: the immunogenic epitope is on the protein, not on the phospholipid. Second, β_2_GPI undergoes a conformational transition upon binding to anionic phospholipids—from a closed circular form to an open J-shaped form, exposing the Arg39–Arg43 epitope on domain I [22,27,28], which is the dominant pathogenic epitope. Third, during pregnancy, phosphatidylserine externalization during cytotrophoblast turnover [29] creates an extensive anionic surface for β_2_GPI in its open conformation, accompanied by a peak of antigen exposure—hence the specific enrichment of anti-β_2_GPI in obstetric APS phenotypes [30,31,32,33].

### 4.3. HAND2 as a Candidate Endometrial Biomarker

The HAND2 finding is coherent with prior data on endometrial-factor infertility. HAND2 has been characterized as a critical mediator of the antiproliferative action of progesterone in the uterus: uterine-specific deletion of HAND2 in mice produces a non-receptive endometrium and embryonic lethality [12]. In humans, reduced endometrial HAND2 expression has been described in endometriosis-associated infertility [13,14], in unexplained implantation failure [15] and in the setting of HAND2-promoter hypermethylation in women with RPL [16]. Our data extend this paradigm to an immune-mediated phenotype: antiphospholipid serology—in our case anti-β_2_GPI—may converge on the same final endometrial pathway.

The clustering of *HAND2* with *IL15* (ρ = 0.65) and *VEGFB* (ρ = 0.70) in our data reproduces the established progesterone-dependent decidualization module [11], which adds biological coherence to the *HAND2* observation beyond a single-gene effect. The categorical cut-off *HAND2* ΔCt ≤ −1.60, with OR 9.62, is a potential starting point for future studies of *HAND2* as a functional biomarker of endometrial receptivity.

### 4.4. Clinical Implications

From the present work, taken together with our previous study [8], three practical implications follow (subject to independent confirmation). First, extended antiphospholipid testing—with priority emphasis on anti-β_2_GPI—is justified in the diagnostic work-up of unexplained RPL. Second, endometrial HAND2 is a candidate functional biomarker linking serology to a molecular endometrial read-out. Third, in anti-β_2_GPI-positive women with evidence of HAND2 deficiency, the mechanistic case for trialing standard APS therapy (low-dose aspirin ± LMWH ± hydroxychloroquine) is strengthened; hydroxychloroquine in particular restores the annexin V shield on trophoblast [21,34] and may be considered in antibody-stratified protocols.

### 4.5. Strengths and Limitations

Strengths: Continuity with the previous serological study [8] ensures a contiguous evidence base from the antibody level to the molecular endometrial phenotype; biopsies were timed synchronously to the cycle phase within the implantation window; the gene panel was biologically coherent and pre-specified; statistical multiplicity was addressed.

Limitations: (i) The subsample size (*n* = 39)—the single-gene *HAND2* association does not survive a stringent Benjamini–Hochberg correction (q = 0.372); the analysis is positioned as hypothesis-generating. (ii) The cross-sectional design does not link *HAND2* expression to a subsequent pregnancy outcome—a critical prospective question. (iii) Progesterone and progesterone-receptor expression were not measured, which would directly test the proposed progesterone-resistance axis. (iv) Antibody testing was performed once, without 12-week confirmation—the same limitation as in [8]. (v) The immunoblot platform is semi-quantitative, with manufacturer-dependent thresholds, and is less standardized than ELISA—this limitation also carries over from [8]. (vi) Single-center recruitment limits generalizability; validation in independent cohorts using ELISA-based methods is required. (vii) Established markers of decidualization such as *FOXO1*, *PRL* and *IGFBP-1* were not assessed; the panel was deliberately restricted to a compact, hypothesis-driven set of receptivity, inflammation and matrix transcripts anchored on *HAND2*, and assessment of these canonical markers is a priority for future targeted validation.

## 5. Conclusions

In women with recurrent pregnancy loss drawn from the previously characterized cohort, the subsample with endometrial biopsy (*n* = 39) almost perfectly preserves the serological profile of the parent 100-patient cohort (Pearson r = 0.999). Among the three antibodies associated with RPL in our previous serological study—anti-prothrombin and anti-annexin V (both non-criteria) and the criterion antibody anti-β_2_-glycoprotein I—only antibodies against β_2_-glycoprotein I were associated, in this exploratory analysis, with reduced endometrial *HAND2* expression, the master transcriptional regulator of stromal decidualization. These exploratory data outline a concrete molecular corridor linking the dominant antiphospholipid specificity (anti-β_2_GPI, a criterion antibody) to a defect in endometrial decidualization and support routine extended antibody testing in RPL as a clinically meaningful continuation of our previous work.

## Figures and Tables

**Table 1:** Baseline clinical characteristics of the molecular subsample (*n* = 39), with rationale for inclusion of each parameter.

Parameter	All Women (*n* = 39)	Rationale for Inclusion
Age, years—Md [IQR]	32 [30–35]	Reproductive age 18–42 (inclusion criterion)
BMI, kg/m^2^—Md [IQR]	22 [21–25]	Normal range for reproductive age
Total pregnancies—Md [IQR]	5 [4–6]	Reproductive burden
Total pregnancy losses—Md [IQR]	2 [2–4]	Consistent with RPL (ESHRE 2023 [1])
Miscarriages < 10 weeks—Md [IQR]	1 [0–2]	Embryonic phase of loss
Miscarriages ≥ 10 weeks—Md [IQR]	0 [0–0.5]	Fetal–placental phase
Missed miscarriages—Md [IQR]	1 [0–2]	Often associated with APL
≥2 pregnancy losses, *n* (%)	34 (87.2)	Inclusion criterion
Endometrial thickness on biopsy day, mm—Md [IQR]	7.4 [6.3–8.0]	Implantation-window standard

Note. Md [IQR]—median and interquartile range; BMI—body mass index.

**Table 2:** Prevalence of antiphospholipid antibodies.

Antibody	Parent Cohort (*n* = 100)	Subsample (*n* = 39)	Class
Anti-β_2_-glycoprotein I	73 (73.0%)	29 (74.4%)	Sydney criterion [6]
Anti-annexin V	43 (43.0%)	16 (41.0%)	Non-criteria
Anti-prothrombin	21 (21.0%)	9 (23.1%)	Non-criteria
Anti-phosphatidic acid	9 (9.0%)	4 (10.3%)	Non-criteria
Anti-phosphatidylcholine	4 (4.0%)	2 (5.1%)	Non-criteria
Anti-phosphatidylglycerol	1 (1.0%)	1 (2.6%)	Non-criteria
Anti-cardiolipin (non-criteria isotype)	<1%	1 (2.6%)	Non-criteria
Others (PI, PS, PE)	0–1%	0 (0%)	Non-criteria
Any panel antibody, *n* (%)	79 (79.0%)	30 (76.9%)	Panel sum

**Table 3:** Endometrial expression (ΔCt, relative log_2_ scale) of the ten panel genes by ncAPL status.

Gene	ncAPL− (*n* = 9) Md [IQR]	ncAPL+ (*n* = 30) Md [IQR]	p (M-W)	p (BH)	r
*HAND2*	−1.30 [−1.56; −0.48]	−2.04 [−2.45; −1.56]	0.037	0.372	−0.47
*VEGFB*	−2.89 [−3.40; −2.54]	−3.19 [−3.68; −2.79]	0.188	0.877	−0.30
*CXCL1*	−6.67 [−6.99; −3.15]	−6.77 [−7.58; −5.36]	0.424	0.877	−0.18
*IFNG*	−12.47 [−13.93; −11.96]	−13.24 [−14.41; −12.16]	0.588	0.877	−0.14
*IL15*	−4.28 [−5.37; −3.58]	−4.70 [−5.22; −3.80]	0.629	0.877	−0.11
*IL8*	−6.92 [−8.58; −3.97]	−7.84 [−8.45; −5.49]	0.661	0.877	−0.11
*TNC*	−3.56 [−4.95; −3.03]	−4.02 [−5.79; −3.02]	0.701	0.877	−0.09
*MMP10*	−11.15 [−11.59; −6.14]	−8.72 [−11.15; −7.56]	0.987	1.000	−0.01
*HPRT1*	−3.73 [−4.07; −3.57]	−3.67 [−3.84; −2.74]	0.594	0.877	+0.12
*C4BPA*	−9.56 [−10.54; −6.12]	−9.37 [−10.14; −8.14]	1.000	1.000	+0.00

Note. Genes are ordered by decreasing absolute effect size r. p (M-W)—two-sided Mann–Whitney U test; p (BH)—Benjamini–Hochberg adjusted; r—rank-biserial correlation (negative values indicate reduced expression in ncAPL-positive women).

**Table 4:** *HAND2* expression.

Antibody	n+	*HAND2* (Ab−)	*HAND2* (Ab+)	*p*	AUC
Anti-β_2_-glycoprotein I	29	−1.39 [−1.94; −0.48]	−2.02 [−2.46; −1.55]	0.064	0.700
Anti-annexin V	16	−1.82 [−2.08; −1.30]	−2.02 [−2.83; −1.55]	0.617	0.549
Anti-prothrombin	9	−1.94 [−2.42; −1.41]	−1.87 [−2.45; −1.55]	0.803	0.470

Note. *HAND2* expression (ΔCt) by each of the three antibodies associated with RPL in our previous serological study [1]. Anti-β_2_GPI is uniquely associated with reduced *HAND2* expression. *p*—two-sided Mann–Whitney U; AUC—area under the ROC curve for *HAND2* as a discriminator of antibody-positive status.

## Data Availability

Experimental and clinical-pathological data is available.
